# *Trichoderma* Strains and Metabolites Selectively Increase the Production of Volatile Organic Compounds (VOCs) in Olive Trees

**DOI:** 10.3390/metabo11040213

**Published:** 2021-03-31

**Authors:** Irene Dini, Roberta Marra, Pierpaolo Cavallo, Angela Pironti, Immacolata Sepe, Jacopo Troisi, Giovanni Scala, Pasquale Lombari, Francesco Vinale

**Affiliations:** 1 Department of Pharmacy, University of Naples Federico II, 80141 Naples, Italy; irdini@unina.it; 2 Department of Agricultural Sciences, University of Naples Federico II, Portici, 80055 Naples, Italy; angela.pironti@unina.it (A.P.); pasquale.lmb@gmail.com (P.L.); 3 Department of Physics “E.R. Caianiello”, University of Salerno, Fisciano, 84084 Salerno, Italy; pcavallo@unisa.it; 4 Institute for Complex Systems, National Research Council, 00185 Rome, Italy; 5 Diagnostica Cavallo S.r.l.—Centro di Ricerca Albo MIUR, 84123 Salerno, Italy; immasepe@gmail.com; 6 Department of Chemistry and Biology “A. Zambelli”, University of Salerno, Baronissi, 84081 Salerno, Italy; jtroisi@unisa.it; 7 Theoreo S.r.l., Montecorvino Pugliano, 84090 Salerno, Italy; info@theoreosrl.com; 8 Department of Veterinary Medicine and Animal Productions, University of Naples Federico II, 80138 Naples, Italy; frvinale@unina.it; 9 Institute for Sustainable Plant Protection, National Research Council, Portici, 80055 Naples, Italy

**Keywords:** *Olea europaea*, biocontrol agents, *Trichoderma*, secondary metabolites, harzianic acid, 6-pentyl-α-pyrone, volatile organic compounds, plant metabolic pathways, GC-MS analysis, Radiello^®^

## Abstract

Plants emit volatile organic compounds (VOCs) that induce metabolomic, transcriptomic, and behavioral reactions in receiver organisms, including insect pollinators and herbivores. VOCs’ composition and concentration may influence plant-insect or plant-plant interactions and affect soil microbes that may interfere in plant-plant communication. Many *Trichoderma* fungi act as biocontrol agents of phytopathogens and plant growth promoters. Moreover, they can stimulate plant defense mechanisms against insect pests. This study evaluated VOCs’ emission by olive trees (*Olea europaea* L.) when selected *Trichoderma* fungi or metabolites were used as soil treatments. *Trichoderma harzianum* strains M10, T22, and TH1, *T. asperellum* strain KV906, *T. virens* strain GV41, and their secondary metabolites harzianic acid (HA), and 6-pentyl-α-pyrone (6PP) were applied to olive trees. Charcoal cartridges were employed to adsorb olive VOCs, and gas chromatography mass spectrometry (GC-MS) analysis allowed their identification and quantification. A total of 45 volatile compounds were detected, and among these, twenty-five represented environmental pollutants and nineteen compounds were related to olive plant emission. *Trichoderma* strains and metabolites differentially enhanced VOCs production, affecting three biosynthetic pathways: methylerythritol 1-phosphate (MEP), lipid-signaling, and shikimate pathways. Multivariate analysis models showed a characteristic fingerprint of each plant-fungus/metabolite relationship, reflecting a different emission of VOCs by the treated plants. Specifically, strain M10 and the metabolites 6PP and HA enhanced the monoterpene syntheses by controlling the MEP pathway. Strains GV41, KV906, and the metabolite HA stimulated the hydrocarbon aldehyde formation (nonanal) by regulating the lipid-signaling pathway. Finally, *Trichoderma* strains GV41, M10, T22, TH1, and the metabolites HA and 6PP improve aromatic syntheses at different steps of the shikimate pathway.

## 1. Introduction

Synthetic pesticides are employed in agriculture to control pests, avoid crop yield losses and product damage. Pesticides may negatively affect human health due to high biological activity and long persistence in the environment [1]. Pesticides in food products and drinking water determine a threat to human health [2]. They accumulate in living species and determine long-term and chronic effects [2]. Fungal biocontrol agents (BCAs) are a natural alternative to control plant diseases, and their complex modes of action generally does not induce resistance in insects, weeds, pests, and pathogens [3]. Microbes of the genus *Trichoderma* are the most studied and marketed fungal BCAs used as active ingredients of various bioformulations in agriculture [4]. They exert a direct activity against pathogens using different mechanisms: antibiosis, parasitism, and competition for nutrients and space [5]. *Trichoderma* spp. have shown rhizosphere competence, may improve plant health and growth, enhance nutrient availability and uptake, induce host resistance, and modify the plant metabolome [6,7]. These positive effects are associated with the production of effector metabolites that selected *Trichoderma* strains can release during the multicomponent interactions with the plant, pathogen, and other microbes [8,9]. For example, *Trichoderma* determines host-induced plant volatile alteration after root colonization in response to the inoculation of different microbial symbioses [10].

Plant volatile organic compounds (VOCs) may act as direct and indirect protective compounds [11,12,13]. They induce metabolomic, transcriptomic, and behavioral responses in receiver organisms, including insect pollinators and herbivores. The composition and concentration of VOCs may influence plant-insect and plant-plant interactions [14]. It is known that olive trees (*Olea europaea* L.) produce VOCs. Some VOCs were characterized in virgin olive oil and processed table olives; among these, C6 compounds, C9 aldehydes, hydrocarbons, and uncharacterized sesquiterpenes were found [15,16,17,18,19]. The olive tree diversifies volatile compounds’ emission depending on the growth environmental conditions, e.g., the water status [20], and the season [21]. Moreover, previous studies showed that *Trichoderma* strains improved the nutraceutical properties of extra virgin olive oil and olive leaves [22,23,24]. Nevertheless, an extensive study on VOCs’ modification in *Trichoderma*-treated olive plants and the possible correlations between the emissions of VOCs and physiological states or interactions with other organisms has not been reported so far. In this work, the effects of *Trichoderma harzianum* strains M10, T22, and TH1, *T. asperellum* strain KV906, *T. virens* strain GV41, or their metabolites harzianic acid (HA) and 6-pentyl-α-pyrone (6PP), on the production of VOCs in olive trees were evaluated. The experiments were conducted in a controlled water regime, as previous studies showed that water availability affects the production of the alcohols, C6-saturated, and unsaturated aldehydes [25]. The VOCs production was monitored by Charcoal cartridge (Radiello^®^) and analyzed by gas chromatography mass spectrometry (GC-MS). The effect of the treatments was evaluated by multivariate analysis to manage the variables and understand their relationships.

## 2. Results

### 2.1. VOCs Identification

In this study, olive trees were treated with *Trichoderma* spore suspensions (*T. harzianum* strains M10, TH1, T22; *T. asperellum* strain KV906, *T. virens* strain GV41) or their metabolite solutions (HA, 6PP) once per month from April to September. Radiello^®^ technology was used to trap the VOCs produced by the plant during the experiments. After seven days of exposure, VOCs were chemically desorbed from the cartridges and analyzed by GC-MS allowing an excellent chromatographic separation. Blank samples were used to subtract noise interferences from sample chromatograms. Traces of benzene were found in blank samples (obtained using unexposed Radiello^®^ samplers) due to its high solubility in carbon disulfide and its ability to be rapidly adsorbed on the activated carbon filter after exposure to the atmosphere. Three technical replicates were performed for each biological sample for a total of 120 GC-MS analyzes. The average of the three technical replicates was considered as the spectrum of the sample (See Supplementary Figure S1). The peaks were then automatically integrated.

The integration, when necessary, was manually corrected and the noise interferences found in blank samples were subtracted from each chromatogram. Forty-five major volatile compounds observed in total ion chromatograms (TICs) were identified by comparing their mass spectra with natural compounds present in database (Table 1).

**Figure 1:**
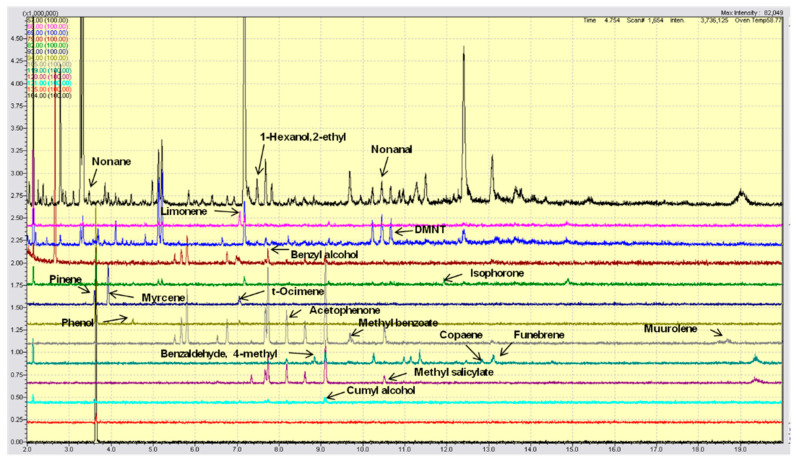
Gas chromatograms of specific olive tree VOCs evaluated using single ion monitoring (SIM) analysis. Different colors are used to better visualize the SIM signals of the VOC compounds (see Table 2), whose peaks are indicated by arrows.

The presence of specific olive tree VOCs was evaluated using single ion monitoring (SIM) analysis (Figure 1). Table 2 shows the nineteen volatile compounds emitted by the olive trees and the relative parameters used for their identification.

### 2.2. VOCs Quantification

The quantification of the VOCs was carried out by integrating the peak areas obtained from the gas chromatographic analysis. The method was validated in terms of linearity (R^2^ ≈ 1), sensitivity (values were within the range established by the limits of detection and quantification), and repeatability (relative standard deviation, RSD, values <15% confirmed the inter-and intraday repeatability). The parameters used to validate the quantification method have been reported as Supplementary Materials (Table S1).

In Table 3, the mean values of the VOCs produced by olive trees following the application of *Trichoderma* strains or metabolites have been reported.

### 2.3. Correlation between Emission of VOCs and Trichoderma Applications

**Figure 2:**
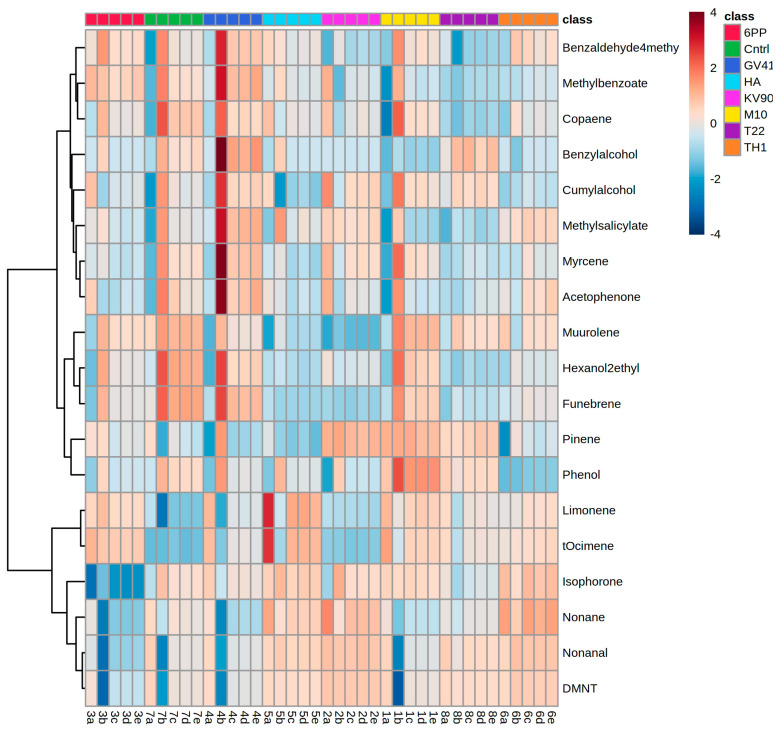
Hierarchical clustering heat map of differential volatile compounds produced by olive trees. Each column represents the samples obtained by plants treated with *Trichoderma* spores (M10, TH1, T22, KV906, and GV41,) or metabolites (6PP and HA). Water-treated plants served as controls (CTRL). In the heat map, red and blue colors indicate higher and lower chemical abundance, respectively. Treatments are indicated according to the color scale (class) shown in the legend at the top right. Data are presented as individual values from each biological replication. Statistical differences were determined using one-way ANOVA (*p* < 0.05).

Replicate samples were grouped according to the abundance of continuous variables in a hierarchical cluster analysis (one-way ANOVA, *p* < 0.05). Figure 2 reports the heat map obtained analyzing the 19 volatile compounds that differentially accumulated among treatments and comparing their chemical abundance vs. control (water-treated plants). Interestingly, all the detected VOCs were significantly affected by the treatments with *Trichoderma* strains or metabolites. Overall, we found that among treated plants, those inoculated with *Trichoderma* strain KV906, HA or 6PP showed a higher number of differential VOCs with lower chemical abundance (blue color), while a higher number of differential compounds with higher chemical abundance (red color) were observed in the control, M10-, GV41-, or TH1-treated plants (Figure 2).

## 3. Discussion

In this study olive trees were treated with *Trichoderma* spore suspensions or their metabolite solutions, and Radiello^®^ technology was used to trap the VOCs [35,36]. Forty-five volatile compounds were detected and identified by GC-MS. These included hydrocarbons (e.g., decane, octane, nonane, etc.) and aromatics (e.g., benzene and derivatives). The most abundant VOCs were benzene, dodecane, toluene, and tetradecane. These compounds are contaminants generally present in the air [26]. Volatile compounds specifically emitted by the olive tree leaves and fruits were obtained by subtracting the contaminants present in the blank sample and comparing the data with the existing literature [27,28,29,30,31,32,33,34,37,38]. VOCs related to the field treatment with biocontrol agents were selected using a targeted metabolomics approach and comparing treated plants with controls. The target VOCs analyses were obtained by extracting the Single Ion Monitoring (SIM) signals from the gas chromatograms acquired in Full Scan function between 40 and 500 *amu/z*. Nineteen compounds, including terpenes, ketones, aldehydes, and aromatic compounds were emitted by plants in response to field treatments with *Trichoderma* spore suspensions or metabolite solutions [39]. Statistical analyses were performed to evaluate the effects of each treatment on the emission of VOCs by olive trees. A hierarchical clustering heat map of differential volatile compounds produced by olive trees was used to understand the optimal method for scaling the data (Figure 2). The search for the selected target VOCs showed a distribution strongly influenced by the treatments. Interestingly, terpenes release was strongly influenced by olive trees’ treatment with strain M10 or the metabolites 6PP and HA. The emission of aromatic compounds was influenced by the application of *Trichoderma* strains T22, TH1, M10, and GV41 or the metabolites (6PP and HA). The emission of aldehydes was mainly affected by strains GV41 and KV906, or the compound HA.

**Figure 3:**
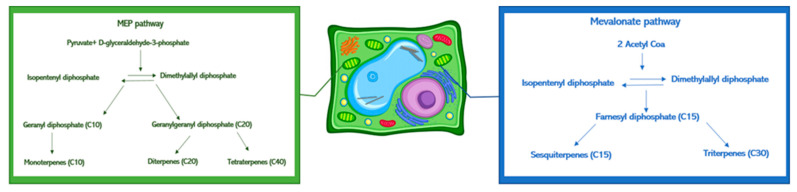
Schematic representation of terpenes biosynthetic pathways in the plant cell.

In the plant cells, the terpenoids’ biosynthesis occurs in the plastids and cytosol (Figure 3). In the plastids, the GDP (prenyl diphosphates geranyl diphosphate) influences the monoterpenes’ synthesis, and the GGDP (geranylgeranyl diphosphate) controls that of the diterpenes. In the cytosol, sesquiterpene biosynthesis occurs using farnesyl diphosphate (FDP) [40]. As already seen in our previous work, these results confirmed *Trichoderma* ability to interfere with the geranyl diphosphate in the biosynthetic pathway [22]. The concentration of myrcene, pinene, and DMNT did not increase after *Trichoderma* treatments. These data can be explained considering that such volatiles are monoterpenes modified into limonene and/or other oxidized metabolites before the plant’s emission. No significant interferences were found with the biosynthesis of sesquiterpenes (copaene, funebrene, and muurolene). Limonene (4-isopropenyl-1-methylcyclohexene) has an important ecological role in plants [41], working as an attractant for pollinators, as part of a defense mechanism, an antifeedant, and an antifungal compound [42]. It is toxic for some herbivore species [43] and is an allelopathic agent in hot and dry climates [44]. Moreover, limonene has chemopreventive, antitumoral [45], hypoglycemic [46], antioxidant [47,48] anti-inflammatory [46], antibacterial [49], and antifungal [50] properties.

**Figure 4:**
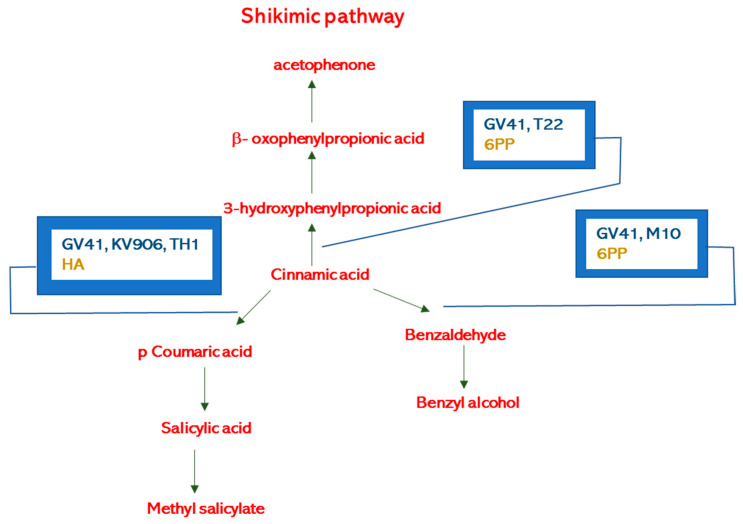
Benzene derivatives biosynthetic pathways and possible influence of *Trichoderma* strains (GV41, T22, M10, KV906, TH1) or metabolites (HA, 6PP).

Elevated levels of nonanal observed in treated plants indicate that biostimulants might modulate the lipid-signaling pathways controlling the enzyme lipoxygenase (LOX). Alkenes and ketones volatile compounds containing C6 to C16 chain are generally the result of fatty acid metabolism [48], while C9-compounds derivate from the oxidation of linoleic and linolenic acid [51,52]. In the lipid-signaling pathway, decarboxylation yields alkanes, methyl ketones, or 1-alkenes, and the carboxy group reduction produces 1-alkanols and aldehydes. Nonanal inhibits *Penicillium cyclopium* and *Botrytis cinerea*’s mycelial growth by disrupting the fungal cell membrane’s integrity, leaking the cell constituents and potassium ions, enhancing the extracellular pH, the total lipid content, and the membrane permeability [53,54]. Moreover, the nonanal acts as an attractant for pollinators [55], is an antifeedant [56], and controls, via the activation of peripheral neurons (e.g., in *Helicoverpa assulta*), the insect oviposition preference [57]. Finally, nonanal has anti-diarrheal properties on humans [58]. The elevated value of methyl salicylate emitted when *Trichoderma* strains GV41, KV906, and TH1, or the metabolites HA and 6PP were used in the field showed that they were able to interfere with the shikimic acid pathway according to previous work [24]. Similarly, it is possible to explain the positive effect of benzyl alcohol release into the environment when *Trichoderma* strains GV41 and M10, and the metabolite 6PP were used in the field (Figure 4) [59].

## 4. Materials and Methods

### 4.1. Fungal Strains and Microbial Metabolites

*T. harzianum* strains M10, T22, and TH1, *T. asperellum* strain KV906, *T. virens* strain GV41 were cultivated as previously described [24]. Fungal spores were collected and maintained at −20 °C in 20% glycerol before use. Spore concentrations were determined by using a hemocytometer. In this work, the *Trichoderma* secondary metabolites harzianic acid (HA) and 6-pentyl-α-pyrone (6PP) were isolated from *Trichoderma* culture filtrate as previously reported [60,61,62].

Fungal metabolites were solubilized in water under continuous stirring overnight.

### 4.2. Plant Material and Experimental Setup

Experiments were conducted on two-years-old olive trees (*Olea europaea* L.) cv. *Carolea*, a typical south Italian variety. The plants were transplanted into pots and placed in a field trial at the Department of Agricultural Sciences at the University of Naples Federico II (Portici, Naples, Italy). The experiments consisted of 8 treatments, including water control, *T. harzianum* strains M10, T22, and TH1, *T. asperellum* strain KV906, *T. virens* strain GV41, and their secondary metabolites harzianic acid (HA), and 6-pentyl-α-pyrone (6PP). The field trial was arranged in a completely randomized block design with a 1.30 m distance between the plants (see Supplementary Figure S2). Cultivar Pendolino was used as a pollinating plant and distributed in the field trial. Olive trees were treated with *Trichoderma* spore suspensions (1 × 10^7^ sp/mL) or fungal metabolite solutions (1 × 10^−5^ M) at the time of transplant by root dip (10 min, 1 L/plant), and repeated every 30 days by soil drenching (400 mL/plant), for a total of 6 applications. Each treatment was applied to five plants with three biological replicates, for a total of 15 plants per treatment.

### 4.3. Collection of VOCs

Charcoal cartridges (Radiello^®^ 130, Supelco, St. Louis, MO, USA) were used to adsorb the olive trees’ VOCs. The cartridge’s adsorbing capacity was about 80 mg, equivalent to exposure to total VOCs of 3000–3500 mg/m^3^ for 8 h or 70,000–80,000 μg/m^3^ for 14 days. The analytical performances of Radiello^®^ were validated by Supelco and reported in the instruction manual. The Radiello^®^ cartridges were placed in the upper part of the olive tree, near the plant’s stem and in a direction parallel to it (see Supplementary Figure S3). All cartridges were set at the same height and were removed on the same day. Five replicates were used per each treatment.

The samples were stored at −20 °C until analysis. The cartridges were analyzed within a week. VOCs were extracted with 2 mL of carbon disulfide (CS_2_) in chlorobenzene (100 µg/L) directly in the Radiello^®^ glass storage tube without drawing out the cartridge. CS_2_ served as an internal standard. The same extraction was carried out on a new unexposed cartridge used as blank. Blank samples were used to subtract noise interferences from sample chromatograms.

### 4.4. GC-MS Analysis

After 30 min, the CS_2_ solution (1 µL) was injected in a GC-MS-QP2010 instrument (Shimadzu Corp., Kyoto, Japan) consisting of a GC-2010 Plus gas chromatograph coupled to a 2010 Plus single quadrupole mass spectrometer. Separations were performed using a 30 m column (JB DB-WAX, 0.25 mm id, 0.25 µm film thickness, Agilent Technologies, Santa Clara, CA, USA), with helium as carrier gas. The initial oven temperature of 40 °C was held for 1 min and then raised to 120 °C at a rate of 5 °C/min, with a further 3 min of hold time. The gas flow was set to achieve a constant linear velocity of 45 cm/s, and the split ratio was set to 10:1. The total run time was 20 min, and the injection temperature was set at 200 °C. The mass spectrometer operated in electron impact (70 eV) in full scan mode in the interval of 40–500 *m/z* with a scan velocity of 5000 *amu/s* and a solvent cut time of 1.9 min. The ion source temperature was set at 200 °C, and the interface temperature at 180 °C. Each biological replicate was run in triplicate (technical replicates) for 120 GC-MS separations. Before the analysis, a solvent blank was analyzed (pure CS_2_ solution), and then an analytical blank was obtained by extracting an unexposed cartridge with 2 mL of CS_2_ solution. Peak areas were automatically normalized to the standard internal area, and metabolite identification was performed by comparing each peak’s mass spectrum with the NIST library collection (NIST, Gaithersburg, MD, USA). The linear index difference max tolerance was set at 10, while the minimum matching for the NIST library search was set at 85%. Using the Kovats index, identification was further confirmed by analyzing a series of hydrocarbons (C10–C26).

Method validation. Calibration curves were built by using external standards. From each curve, it was possible to obtain the linearity (from the regression coefficient), the limit of detection (LODs = 3 × $\frac{\mathrm{standard}\;\mathrm{deviation}}{\;angular\;coefficient}$), and quantification (LOQs = 10 × $\frac{\mathrm{standard}\;\mathrm{deviation}}{\;angular\;coefficient}$). Intraday repeatability was tested by injecting eight different concentrations of each standard three times. The interday variations were found carrying out after seven days of the same experiments. Standards were purchased by Sigma-Aldrich (St. Louis, MO, USA) except Muurolene (Molbase Biotechnology Co., Ltd. Shanghai, China).

### 4.5. Statistical Analysis

Results were summarized in a comma-separated matrix (CSV) file and loaded in the appropriate statistics manipulation software. The normalization procedures consisted of data transformation and scaling. Data transformation was made by generalized log transformation and data scaling by autoscaling (mean-centered and divided by each variable’s standard deviation) [63]. The chromatographic data were tabulated with one sample per row and one variable (metabolite) per column. Statistical analysis was performed using Statistica software (StatSoft, Tulsa, OK, USA) and Minitab (Minitab Inc., State College, PA, USA). Fisher’s least significant difference (LSD) post hoc test at the 0.05 level of significance was used.

Clustering analysis. Metabolite expression ratios of VOCs (column) were log2 transformed and normalized using z-scores. In the vertical heat map, colour coding indicated concentration differences for each metabolite (row): z-scores >0 (red), <0 (blue) and ≈0 (white).

## 5. Conclusions

This study allowed the analysis of the volatile organic substances emitted by olive trees under treatment with formulations based on different strains belonging to the genus Trichoderma or metabolites extracted from them. Forty-five compounds have been identified by gas chromatography, of which nineteen compounds were imputable to olive trees emissions. Statistical analysis showed that each treatment influenced the different VOCs’ relative concentrations, allowing to obtain a characteristic profile of compounds released by the olive tree treated with *Trichoderma* living fungus or its metabolites. For these compounds each treatment’s relative concentrations were explored to allow a quick and visual interpretation of the results obtained. Considering relevant metabolic pathways acting on VOCs’ formation after treatments, we found a significant effect on three biosynthetic pathways: methylerythritol 1-phosphate (MEP) pathway (by *Trichoderma* strain M10, and metabolites 6PP and HA), lipid-signaling pathway (by *Trichoderma* strains GV41, KV906 and the metabolite HA) and shikimate pathway (by *Trichoderma* strains GV41, M10, T22, TH1, and the metabolites HA and 6PP) (Table 4).

Considering that the different volatile compounds have different activities and roles in the ecosystems, our work provides essential information on olive tree/*Trichoderma* interaction. This interaction positively interferes with VOCs’ biosynthesis, confirming the beneficial effects of these microbes on plants.

## Figures and Tables

**Table 1:** List of olive tree VOCs identified in this work. Radiello^®^ technology was used to trap the VOCs during the experiments. After seven days of exposure, VOCs were chemically desorbed from the cartridges and analyzed by GC-MS. The asterisk (*) indicates air contaminants [26].

N.	Compound Name	N.	Compound Name
1	1-Heptene, 2,6,6-trimethyl-	24 *	Ethylbenzene
2	1-Hexanol, 5-methyl-2-(1-methylethyl)-	25 *	Hexadecane
3	2,3-Dihydroxystearic acid	26 *	Hexane, 3,3,4-trimethyl-
4	3-Thiazolidinecarboxylic acid, 4-(acetyloxy)-2-(1,1-dimethylethyl), phenylmethyl ester, 1-oxide, [1R-(1.α., 2.β.,4.β)]	27	Isobutyl 2-methylpentyl carbonate
5	Acetic acid, butyl ester	28	Methylene chloride
6 *	Benzene	29	Nonane, 2,3-dimethyl-
7 *	Benzene, 1,2,4-trimethyl-	30	Nonane, 2,5-dimethyl-
8 *	Benzene, 1,3-bis(1,1-dimethylethyl)-	31	Nonane, 5-(2-methylpropyl)-
9 *	Benzene, 1,3-dimethyl-	32 *	Octane, 1,1’-oxybis-
10 *	Benzene, 1-ethyl-4-methyl-	33 *	Octane, 5-ethyl-2-methyl-
11	Butyronitrile, 2-(trimethylsilyloxy)-(3S)-(t-butoxycarbonyl)amino-	34 *	*o*-Xylene
12	Cyclohexene, 1-methyl-4-(1-methylethenyl)-, (S)-	35 *	*p*-Xylene
13 *	Cyclopentane, (2-methylpropyl)-	36	Sulfurous acid, butyl nonyl ester
14 *	Decane, 1-iodo-	37	Sulfurous acid, decyl hexyl ester
15 *	Decane, 2,3,5,8-tetramethyl-	38 *	Tetrachloroethylene
16 *	Decane, 2-methyl-	39	Tetradecane
17 *	Decane, 3,7-dimethyl-	40	Tetradecane, 5-methyl-
18 *	Decane, 3,8-dimethyl-	41 *	Toluene
19 *	Decane, 4-methyl-	42	*trans*-2,3-Epoxydecane
20 *	Decane, 5-methyl-	43 *	Tridecane
21 *	Dodecane	44 *	Undecane
22 *	Dodecane, 4,6-dimethyl-	45	Undecane, 6-ethyl-
23	Ethanol, 1-(1-cyclohexenyl)-		

**Table 2:** List of specific olive tree volatile compounds found in this study and the parameters used for their identification.

N.	Compound	Class	CAS Number	SIM Signal (*amu/z*)	Kovats Index	Ref.
1	1-Hexanol, 2-ethyl-	Alcohols	104-76-7	57	1030	[27]
2	Acetophenone	Aromatic ketones	98-86-2	105	1068	[28]
3	Benzaldehyde,4-methyl-	Aromatic aldehydes	104-87-0	119	1086	[16]
4	Benzyl alcohol	Alcohols	100-51-6	79	1040	[27]
5	Copaene	Sesquiterpenoids	3856-25-5	119	1375	[28]
6	Cumyl alcohol	Alcohols	617-94-7	121	1084	[29]
7	DMNT [(E)-4,8-Dimethyl-1,3,7-nonatriene]	Terpenoids	51911-82-1	69	1759	[30]
8	Funebrene	Terpenoids	50894-66-1	119	1403	[31]
9	Isophorone	Cyclic ketones	78-59-1	82	1123	[32]
10	Limonene	Monoterpenes	138-86-3	68	1030	[28]
11	Methyl benzoate	Benzoic acid esters	93-58-3	105	1096	[28]
12	Methyl salicylate	Benzoic acid esters	119-36-8	120	1192	[28]
13	Muurolene	Sesquiterpenoids	10208-80-7	105	1497	[28]
14	Myrcene	Monoterpenes	123-35-3	93	991	[31]
15	Nonanal	Aldehydes	124-19-6	57	1107	[28]
16	Nonane	Hydrocarbons	111-84-2	57	900	[33]
17	Phenol	Phenols	108-95-2	94	1011	[27]
18	Pinene	Monoterpenes	2437-95-8	93	943	[34]
19	t-Ocimene	Monoterpenes	13877-91-3	93	976	[30]

**Table 3:** Quantification of the VOCs produced by olive trees following the application of *Trichoderma* strains (M10, T22, TH1, KV906, GV41) or metabolites (HA, 6PP). Symbols in the tables indicate statistical differences among treatments as follows: * indicates a *p*-value < 0.05 compared to CTRL; § indicates a *p*-value < 0.05 compared to 6PP; ¥ indicates a *p*-value < 0.05 compared to GV41; ¶ indicates a *p*-value < 0.05 compared to M10; & indicates a *p*-value < 0.05 compared to T22; % indicates a *p*-value < 0.05 compared to TH1; £ indicates a *p*-value < 0.05 compared to KV906; @ indicates a *p*-value < 0.05 compared to HA. Each symbol has been also repeated under the treatment of reference in the first line of the Table.

VOC (*p*-Value)	CTRL *	M10	T22 &	TH1 %	KV906 £	GV41 ¥	HA @	6PP §
1-Hexanol, 2-Ethyl- (0.0013527)	18.35 ± 4.29	16.03 ± 4.59	10.66 ± 0.18 §*¶	11.00 ± 0.75 §*¥¶	11.42 ± 0.30 §*¶	15.45 ± 4.95	10.58 ± 0.47 §*¶	15.70 ± 4.25
Acetophenone (5.03 × 10^−1^)	11.88 ± 0.99 §¥	11.53 ± 1.03 §¥	12.00 ± 0.52 §¥	11.32 ± 0.62 §¥	11.06 ± 0.42 §¥	13.14 ± 0.87 *	11.40 ± 0.24 §¥	13.47 ± 0.88 *
Benzaldehyde, 4-Methyl (1.11 × 10^−2^)	2.96 ± 0.49 §¥	3.17 ± 0.36 §&	2.70 ± 0.18 §¥¶	2.90 ± 0.20 §¥&	2.46 ± 0.33 *§¥¶&	3.42 ± 0.37 *§£&	3.08 ± 0.06 §&	3.93 ± 0.32 *£%
Benzyl alcohol (1.51 × 10^−5^)	3.68 ± 0.16 §&	3.17 ± 0.10 *§¥@&	4.32 ± 0.40 *	3.13 ± 0.24 *§¥@&	3.21 ± 0.16 *§¥&	4.35 ± 0.44 *	3.53 ± 0.31 §¥	4.18 ± 0.19 *
Copaene (0.031723)	2.35 ± 0.62 *	2.19 ± 0.69	1.71 ± 0.03 §¥	1.77 ± 0.16 §*	1.88 ± 0.18 §	2.25 ± 0.32	2.06 ± 0.17	2.46 ± 0.34
Cumyl alcohol (0.00047929)	1.60 ± 0.28 §&	1.79 ± 0.27	1.91 ± 0.18	1.38 ± 0.03 §¥£¶&	1.73 ± 0.16	1.85 ± 0.22 %	1.42 ± 0.20 §¥£¶&	1.92 ± 0.23
DMNT (0.00010625)	7.00 ± 4.43 £&%	7.18 ± 4.78 £&%	11.10 ± 0.64 *	12.53 ± 0.81 *	14.14 ± 0.47 *	6.12 ± 3.99 @£&%	10.50 ± 0.23	4.95 ± 3.19 @£%
Funebrene (3.14 × 10^−2^)	6.68 ± 1.48 ¶&%£@§	5.46 ± 1.22 &%£¥@	3.64 ± 0.55 *£¥§	3.82 ± 0.09 *	2.79 ± 0.10 *¶¥§	6.08 ± 1.68 &%@	3.14 ± 0.10 *¥§	5.08 ± 1.44 *&£@
Isophorone (2.35 × 10^−2^)	2.16 ± 0.28 @§§	2.46 ± 0.20 &	1.98 ± 0.14 ¶%	2.56 ± 0.29 &	2.19 ± 0.64	2.12 ± 0.51 @§	2.66 ± 0.21 *&£¥§	1.12 ± 0.20 *¶&%£¥@
Limonene (3.93 × 10^−2^)	3.30 ± 0.75 ¶&%¥@§	5.45 ± 0.76 *£	5.00 ± 0.27 *£@§	4.56 ± 0.16 *@§	3.64 ± 0.15 ¶&@§	4.43 ± 1.42 *@§	6.42 ± 1.56 *&%£¥	6.32 ± 0.22 *&%£¥
Methyl benzoate (3.54 × 10^−3^)	5.10 ± 0.98 %¥§	4.57 ± 0.85 §	4.39 ± 0.13 §	4.16 ± 0.17 *&§	4.43 ±0.68 ¥§	6.20 ± 0.87 *¶&%£@	4.89 ± 0.37 ¥§	6.80 ± 0.21 *¶&%£@
Methyl salicylate (0.00071484)	2.58 ± 0.36 ¥§	2.37 ± 0.25 ¥§	2.47 ± 0.26 ¥§	2.68 ± 0.05 ¥§	2.70 ± 0.12 ¥§	3.17 ± 0.31 *¶£@	2.72 ± 0.48 ¥	3.06 ± 0.06 *¶&%£
Muurolene (3.98 × 10^−1^)	3.09 ± 0.72 &%£¥@	3.17 ± 1.65 &%£¥@	1.81 ± 0.70 *¶£@	1.54 ± 0.64 *¶&£	0.32 ± 0.06 *¶&%§	1.40 ± 0.74 *¶	0.66 ± 0.31 *¶&§	2.17 ± 1.09 £@
Myrcene (0.00088287)	11.66 ± 1.23 ¥§	12.14 ± 1.64 @	11.48 ± 0.65 ¥	10.42 ± 0.42 ¶¥	11.41 ± 0.42 ¥	13.22 ± 1.75 *&%£@§	10.70 ± 0.43 ¶¥§	13.02 ± 0.31 *&%£@
Nonanal (1.39 × 10^−1^)	9.20 ± 5.59 &%£@	8.67 ± 5.27 &%£@	14.30 ± 1.90 *¶¥§	16.44 ± 1.02 *¶§	17.15 ± 0.37 *¶¥§	8.57 ± 4.88 &%£@	15.08 ± 1.15 *¶¥§	5.33 ± 2.98 &%£@
Nonane (7.07 × 10^−2^)	3.43 ±0.58	2.91 ±0.65 @%	3.57 ±0.06 ¥£%	4.84 ±0.57 *§¥	4.47 ±0.59 *§¥	2.63 ±1.01 @	4.37 ±0.46 *§	2.75 ±0.83
Phenol (2.01 × 10^−4^)	1.10 ± 0.11	1.59 ± 0.17 §*¥@	1.22 ± 0.02 ¥@¶	0.65 ± 0.02 §*¥@¶&	0.83 ± 0.24 §*¶&	0.96 ± 0.16	0.99 ± 0.24	1.08 ± 0.17
Pinene (1.28 × 10^−6^)	3.03 ± 0.44 §	3.78 ± 0.22 *¥@£%	3.79 ± 0.16 *¥@£%	2.83 ± 0.20 §£	3.51 ± 0.19 §*¥@	2.93 ± 0.11 §	2.91 ± 0.06 §	3.88 ± 0.06
t-Ocimene (1.13 × 10^−2^)	1.94 ± 0.10 §	3.51 ± 0.76 §*£	3.16 ± 0.19 §*@£	2.98 ± 0.09 §*£	1.97 ± 0.09 §@	2.86 ± 0.85 §*@£	4.02 ± 1.21 *	4.29 ± 0.13

**Table 4:** Olive VOC biosynthetic pathways possibly affected by *Trichoderma* applications (strains or metabolite).

*Trichoderma*	Metabolic Pathway	Class of VOCs
Strain GV41	Shikimate pathway	Aromatics
	Lipid-signaling pathway	Aldehydes
Strain KV906	Lipid-signaling pathway	Aldehydes
Strain M10	MEP pathway	Terpenes
	Shikimate pathway	Aromatics
Strain T22	Shikimate pathway	Aromatics
Strain TH1	Shikimate pathway	Aromatics
Metabolite HA	MEP pathway	Terpenes
	Shikimate pathway	Aromatics
	Lipid-signaling pathway	Aldehydes
Metabolite 6PP	MEP pathway	Terpenes
	Shikimate pathway	Aromatics

## Data Availability

The data presented in this study are available on request from the corresponding author or the first author. The data are not publicly available due to privacy.

## Supplementary Materials

The following are available online at https://www.mdpi.com/article/10.3390/metabo11040213/s1, Table S1: Parameters used to validate the relative-quantification method of the VOCs produced by olive trees following the application of Trichoderma strains or metabolites, Figure S1: Example of an integrated gas chromatogram resulting from the extraction of plant VOCs sampled by Radiello® cartridges, Figure S2: Randomized block design of field experiment, Figure S3: Application of the Radiello® cartridges to olive trees.
