# Supplementary material for: Trichoderma Strains and Metabolites Selectively Increase the Production of Volatile Organic Compounds (VOCs) in Olive Trees
Source: Metabolites. 2021 Mar 31;11(4):213. doi: 10.3390/metabo11040213 (PMC8066342; doi:10.3390/metabo11040213)
Supplement: Supplementary file 1 [file metabolites-11-00213-s001.pdf]

Supplementary materials

Table S1. Parameters used to validate the relative-quantification method of the VOCs produced by olive trees following the application of *Trichoderma* strains or metabolites. Data include compound name (VOC), retention time (Rt, min), calibration curve and R^2, limits of detection and quantification (LOD and LOQ, respectively), Intraday and Interday repeatability expressed as relative standard deviation (RSD %) on 3 replicates (n=3).

VOC	Rt (min)	Calibration curve	R^2	LOD	LOQ	Intraday RSD% (*n* = 3)	Interday RSD% (*n* = 3)
**Hexanol, 2-ethyl**	7.6	y = 4,9x + 5,4	0.9988	3.00	10.00	1.8	2
**Acetophenone**	8.2	y = 2x + 8	1	3.00	10.00	0.7	1
**4-Methyl-Benzaldehyde, 4-Methyl**	9.0	y = 1,15x + 0,9667	0,9994	3.54	11.51	0.9	1.2
**Benzyl alcohol**	7.8	y = 1,25x + 1,4667	0.995	3.00	10.00	2.1	2.4
**Copaene**	13.0	y = 0,7x + 0,2667	0.9932	3.00	10.00	0.5	1.0
**Cumyl alcohol**	9.15	y = 1,05x - 0,1667	0.9643	3.06	10.18	1.4	1.3
**DMNT**	10.6	y = 6,75x - 0,3333	0.9995	3.00	10.00	1.8	1.7
**Funebrene**	13.2	y = 2,45x + 0,0667	0.9999	3.00	10.00	0.9	1.2
**Isophorone**	12.0	y = 2x - 1,2	0.9709	3.04	10.15	1.7	1.9
**Limonene**	7.0	y = 2,25x + 0,3333	0.9959	1.33	10.02	2.1	2.4
**Methyl benzoate**	9.8	y = 2,4x + 1,5333	0.9977	3.00	10.00	1.1	1.4
**Methyl salicylate**	10.4	y = 1,2x + 0,7667	0.9977	3.00	10.00	1.3	1.5
**Muurolene**	18.8	y = 1,3x - 0,2	0.9826	3.03	10.08	2.0	1.8
**Myrcene**	4.0	y = 5,75x - 3	0.9514	3.07	10.25	2.0	1.8
**Nonanal**	10.5	y = 6,75x - 2,9333	0,9702	3.04	10.15	0.9	1.1
**Nonane**	3.4	y = 1,3x + 0,7	1	3.00	10.00	0.5	0.7
**Phenol**	4.6	y = 0,4x + 0,05	0.9552	3.07	10.23	3.0	2.5
**Pinene**	3.6	y = 3,05x - 1,8333	0.9748	3.04	10.13	1.5	1.8
**t-Ocimene**	7.15	y = 1,5x	1	3.00	10.00	3.0	2.7

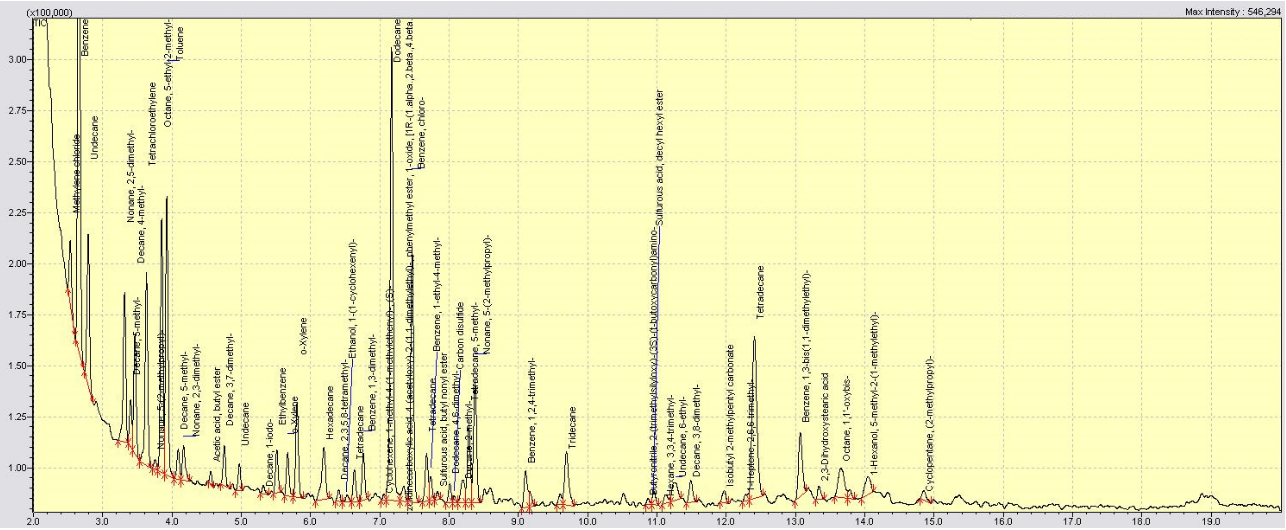

Figure S1. Example of an integrated gas chromatogram resulting from the extraction of plant VOCs sampled by Radiello® cartridges.

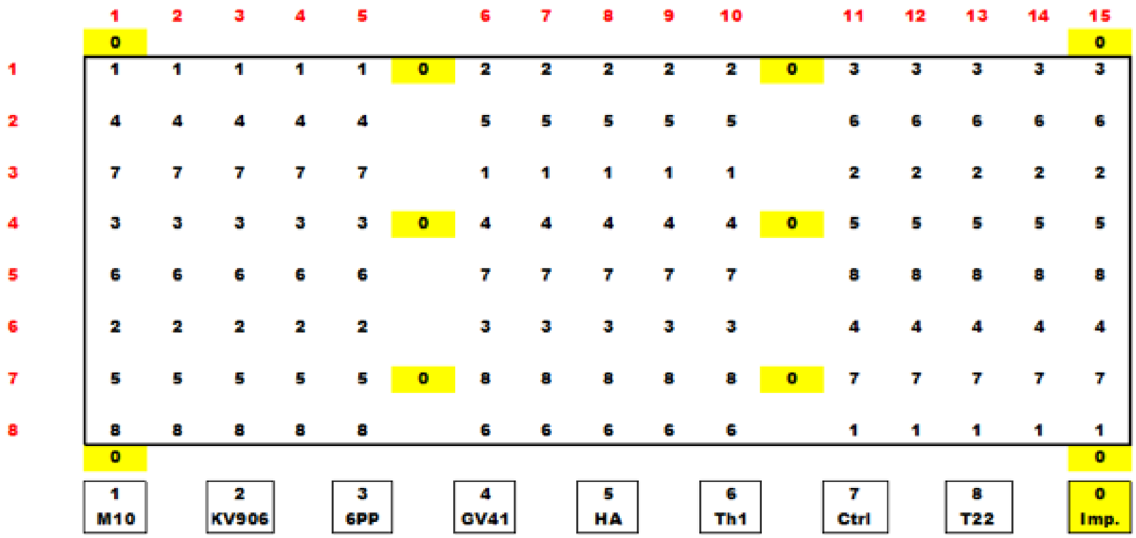

Figure S2. Randomized block design of field experiment. Treatments: *Trichoderma harzianum* strains M10, T22, and TH1, *T. asperellum* strain KV906, *T. virens* strain GV41, harzianic acid (HA) and 6-pentyl-α-pyrone (6PP). Numbers in the figure represent the treatments as indicated in the boxes below the scheme. The following parameters were used: N° of plants per treatment: 15; Plant layout: 1.30m x1.30m plant-plant; concentration of *Trichoderma* spore suspensions: $1*10^7$ spore/mL; concentration of *Trichoderma* metabolite solutions:$1*10^{-5}$ M.

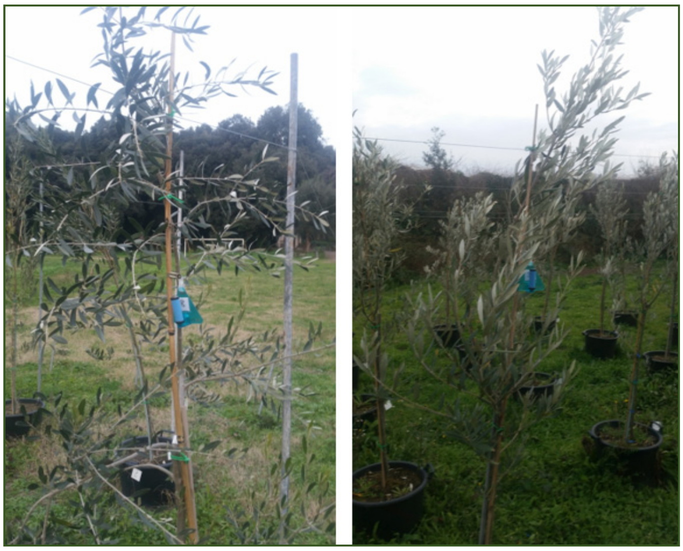

Figure S3. Application of the Radiello® cartridges to olive trees.
